# Challenges to the performance of the regional health departments in Mauritania: a qualitative study

**DOI:** 10.1186/s12913-025-12625-9

**Published:** 2025-04-16

**Authors:** Mohamed Ali Ag Ahmed, Kirsten Accoe, Karina Kielmann, Bruno Marchal, Verónica Trasancos Buitrago, Hamma Issa Moussa, Bart Criel

**Affiliations:** 1 Institute of Tropical Medicine, Antwerp, Belgium; 2 Department of Management, Evaluation and Health Policy, School of Public Health, Université de Montréal, Montreal, Canada; 3 Enabel, Brussels, Belgium

**Keywords:** Regional level, Intermediary level, Health system, Performance, Mauritania, Sub-Saharan Africa

## Abstract

**Background:**

In sub-Saharan Africa, the regional or intermediary level should play an important role within the health system. It is generally responsible for providing technical support to the health districts, ensuring the contextualisation of policies, and channeling communication between the central and local levels. In this paper, we analyze factors influencing the performance of the two Regional Health Directorates in Mauritania. More specifically, we analyse the main challenges they face and recommend actions to strengthen their performance.

**Methods:**

A case study approach with a qualitative study design was used. Two Regional Directorate for Health Action (RDHAs) of Brakna and Nouakchott-North were chosen as units of analysis. The study was conducted from February to August 2021 and guided through the conceptual framework of Marchal et al. and triangulation of a document review, focus group discussions and semi-structured individual interviews. A total number of 25 respondents were purposively selected, covering the main stakeholders who manage or collaborate with the RDHAs, until saturation and a certain level of diversification was reached. Thematic content analysis and aided by Nvivo software were used. A mixed (inductive and deductive) approach to analysis was adopted.

**Results:**

This study assessed the performance of two RDHAs in Mauritania by analysing of their key functions. The participants noted a lack of clarity in their objectives, an overly bureaucratic vision of their functions and inadequate technical capacities hampering the provision of required services. They mentioned weak regional health coordination with stakeholders and a poor organisational culture. These bottlenecks largely limited the capacities of the RDHAs to play their role within the health system in Mauritania.

**Conclusion:**

This study identified the main gaps in the performance of the Brakna and Nouakchott-North RDHAs. The scale of these challenges does not allow them to engage and mobilise the right stakeholders and appropriate resources to achieve their objectives. We propose to initiate organisational change at the RDHA level. Some actions are discussed.

## Background

Several low- and middle-income countries (LMICs) have included a degree of decentralisation of their health systems as part of broader reforms. In sub-Saharan Africa (SSA), this decentralisation has often involved a reorganisation of health systems into three levels: central, intermediate and local. These three levels are differentiated on the one hand by their functions and on the other hand by the governance means that attribute variable forms of authority to them [1]. There is ample literature on the functions of central and local levels of health systems. The central (national) level is associated with the definition of national policies and plans and the allocation of resources, while the local (district or equivalent) level makes them operational. In contrast, the role of the intermediate level (e.g. regions, provinces, states or zones) appears less clearly defined. While in principle responsible for providing technical support to the health districts, ensuring the contextualisation of policies, and channeling communication between the central and local levels. In practice, its effectiveness is limited due to several challenges, including regulatory inconsistencies, unclear policies, resistance to change, weak management capacity, centralisation of key resources, and weak monitoring and support capacity at the central level [1, 2].

Mauritania is an essentially desert country with a large coast at the Atlantic ocean, and bordering Morocco in the North, Mali in the South-East and Senegal in the South. It has a surface area of 1,030,700 km^2^ for an unevenly distributed population estimated at 4,173,077 inhabitants (in 2020). With a density of 4.5 inhabitants per km^2^, it is the fourth least densely populated country in Africa. In 2010, the government’s adoption of the " Decentralization and Local Development Policy Declaration” reaffirmed the political will of the public authorities to make decentralisation an irreversible choice. This decentralisation policy has given rise to a health system whose organisation is based on a pyramidal administrative structure divided into central, intermediate and departmental levels (the latter being referred to as *Moughataa* health district [MHD], equivalent to the notion of “health district”). The Mauritanian health system is marked by institutional instability, with recurrent turnover of health ministers and central directors. The intermediate level is constituted by the Regional Directorate for Health Action (RDHAs), whose role and organisation have been established by Decree 178/2016/MoH, and headed by a director assisted by six heads of social and health services appointed by the order of the Minister of Health. The RDHAs are placed under the hierarchical authority of the *Wali* (equivalent to the regional governor, within the Ministry of Interal Affairs). They appear to face several challenges, some of which are specific to them, while others are common to the rest of the health system. A more precise identification of these challenges through an analysis of their performance will enable the RDHAs to design strategies and implement actions to fully play their role within the health system.

The Institutional support for the health sector support program (AI-PASS) is funded by the European Union and implemented by ENABEL, the Belgian Development Agency, in collaboration with the Institute of Tropical Medicine (ITM) in Antwerp. It aims, through support to the Ministry of Health (MOH), to strengthen the health system. At the national level, the program supports the MOH in five main areas, which follow the national health development plan: governance, health care provision, human resources, health financing and medicines & consumables. At the local level, this support is provided through an action-research approach in two learning sites “moughataas”, Dar Naim and Bababé, which are under the authority of the RDHAs of Nouakchott-Nord and Brakna respectively [3–5]. The regional level has received less attention in this programme, although it also faces challenges that affect the overall performance of the health system. The importance of identifying and better understanding these challenges were recognised during the first phase of the AI-PASS program. Addressing them will strengthen the synergy between the three levels of the health system. This analysis is therefore part of the AI-PASS program’s support to the MOH.

In this paper, we analyse factors influencing the performance of the two RDHAs in Nouakchott-Nord and Brakna in Mauritania. More specifically, we analyse the main challenges or bottlenecks that RDHAs face and recommend actions to strengthen their performance within the Mauritanian health system.

## Methodology

We use a case study approach [6] with a qualitative study design. Case studies are recognized for their relevance when it comes to studying phenomena in depth in a real situation in their context [7]. We consider the case being the RDHA level of the health system. An informed choice was made regarding the two units of analysis: the RDHAs of Brakna, located in the centre of the country, and Nouakchott-Nord within the capital, where the AI-PASS Program intervenes. The study was conducted over seven months (February to August 2021).

### Analytical framework

**Fig. 1:**
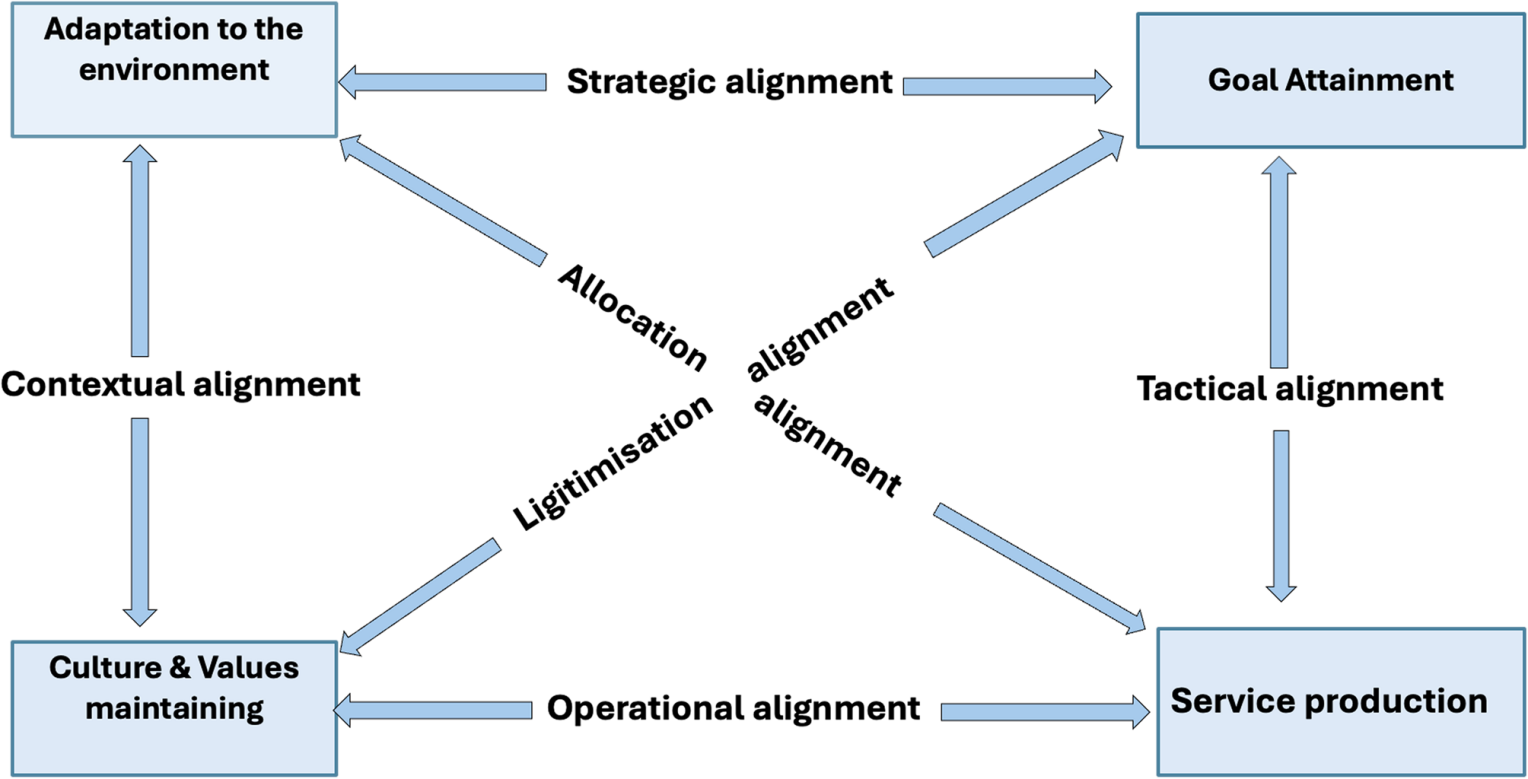
The multipolar framework of Marchal et al. [8]

We used the organisational performance model of Marchal et al. [8] (Fig. 1), adapted from Sicotte & al. [9]. This model, whose scope is extended to support health care organisations (as are the RDHAs), embraces the complexity of organisations. It allows for a dynamic and iterative analysis. The model stipulates four essential functions that an organisation must constantly maintain in synergy. These include: 1. goal attainment; 2. service production; 3. Maintenance of culture & values; and 4. adaptation to the environment. These four functions interact with each other, a relationship which the authors refer to as ‘alignments’ [8].

### Data collection

This paper draws on the triangulation of three data sources: a document review and group and individual interviews.

*The document review* covered reports and documents produced by the Ministry of Health (MOH), the RDHAs, and other actors (as technical and financial partners) within the framework of the AI-PASS Program. These were used to guide the design of the interview guides.

*Three focus groups* were conducted with key stakeholders selected by the first author for their knowledge, experience and involvement in health policies at the regional level in Mauritania. A total of 25 people participated in the focus groups, including 7 women and 18 men, all over the age of 25 (see Table 1). In order to have homogeneous groups, Two groups of nine and eight people were made up of participants from both regions. The third group of eight people was constituted by the two RDHAs, two staff from the central level and from four international technical and financial partners (TFPs). These focus groups lasted approximately 90 min and were facilitated by the first author. Notes were taken of these focus groups and helped guide the design of the individual interview guides.

Finally, *semi-structured individual interviews* were conducted with 20 participants selected in a reasoned way among those who had participated in the focus groups (see Table 1). This technique allows the researcher to rely on his or her own judgement to identify and select the participants with the most information about the phenomenon of interest [10–12]. The list of participants was drawn up at the various workshops organised by the AI-PASS project. Their number and characteristics were guided by the criteria of empirical saturation and diversification [11, 13]. The interviews were conducted by the lead researcher. They were conducted in French, recorded and lasted between 35 and 118 min.

Flexible guides were designed, tested, and amended before data collection began for both FGDs and individual interviews. Their content focused on the main challenges or obstacles to RHDC’s performance in relation to its four functions: the achievement of objectives, the production of services, interactions with stakeholders, and organisational culture and values.

**Table 1:** Characteristics of participants in focus groups and semi-structured individual interviews

Level/function in the health system	Individual interviews	Focus groups		
		Group 1	Group 2	Group 3
RDHAs Nouakchott-North and Brakna	2 regional directors and their 6 heads of department	03 heads of department	03 heads of department	2 regional directors
Ministry of Health (central level)	2 Central Directors			2 Central Directors
Moughataas	02 Chief Doctors	01 Chief Doctor	01 Chief Doctor	
Wilaya (region)	01 Health Advisor to the Wali	01 Health Advisor to the Wali		
Ministry of Social Affairs, Childhood and Family (MSACF)			01 MSACF regional coordinator	
Regional Councils	02 Regional coordination officers	02 Regional coordination officers	02 Regional coordination officers	
Technical and financial partners (TFP)	03 TFP representatives			04 TFP representatives
Private sector		01 Doctor		
Hospitals	02 Hospital Directors	01 Hospital Director	01 Hospital Director	
**Total (participants)**	**20**	**9**	**8**	**8**

### Data analysis

A thematic content analysis was conducted [14, 15]. The individual interviews were transcribed verbatim. These were then imported into the Nvivo software. A mixed (inductive and deductive) approach to analysis was then adopted. The verbatim transcripts were examined line by line to generate codes in the form of labels attached to analysis units of varying sizes. The closest codes were then grouped under sub-themes and then under four themes that correspond to the functions suggested by our analytical framework [8]. This approach made it possible to obtain a tree with thematic sets (convergent, divergent or sometimes complementary) constituting types of meaning matrices that coexist with each other. These have thus made possible to link the challenges to the different functions of the RDHA and consequently appreciate the performance of the RDHA.

In addition, the detailed notes taken during the documentary review and the contents of the group interviews were a useful source of information contributing to validating or contrasting the data from the individual interviews.

## Results

The main results of individual interviews in this study have been grouped according to the four functions proposed by Marchal et al. [8] to assess organizational performance, in this case, the RDHAs in Mauritania. The nature and intensity of interactions and alignment between these functions are studied in more detail.

### Goal attainment: the objectives of the RDHAs

The first group of participants stated that the RDHA’s objective is to improve access to and quality of curative, preventive, and promotional health services.

*“I see the RDHA as having the objective of providing quality care to the people of the Wilaya. It must bring health services to the most remote areas*,* ensure the availability of medicines*,* raise awareness against diseases…” (Staff*,* RDHA).*

The second group of participants, however, considered this objective to be of a more operational nature, and would therefore be within the scope of goals of the level of the Moughataas (i.e. the district level). For this second group, the main objective of RDHA would rather be to ensure the implementation at the regional level of the MOH health policies and coordinate multisectoral action:

*“The objective of the RDHA is the execution of the MOH policy in its Wilaya and maybe the collaboration with other sectors that are not health” (Staff*,* RDHA).*

*“For us*,* the RDHA must pilot all health and social actions at the regional level. It represents the Ministry of Health. It is the focal point for health at the regional level” (Hospital Director).*

However, some participants felt that this objective was too ambitious. They consider that the RDHAs do not have the levers or the means to achieve this objective.

*“This objective is very broad. The RDHA does not have the means to achieve it*,* which is why they focus on health services and not on the coordination of health action at the regional level*,* which limits their scope of action.” (TFP).*

### Service production: delivery of services offered by the RDHAs and operational management

#### Services offered by the RDHA

The main services identified by most participants were training, supervision and planning of activities.

##### Staff training

For several participants, the RDHA should play a role in training their own staff and that of the Moughataas. They noted, however, that the RDHA did not have a training plan and that the trainings offered were organised by the central level or by Technical and Financial Partners (TFPs). Some participants mentioned that these training sessions do not always correspond to their priority needs.

*“At the RDHAs level*,* we have a pool of trainers for the various components*,* whether reproductive health*,* tuberculosis*,* nutrition or vaccination*,* but we do not have the means to capitalise training for the Moughataas to be able to strengthen their skills” (Staff*,* RDHA).*

On the other hand, other participants indicated that:

*“The RDHA team is not trained enough; this is a weakness of the RDHA…. And when we ask to do the cascade training it doesn’t go well… Therefore we see this substitution of the RDHA by the central level to provide this training” (Central level).*

Furthermore, some participants observeed that even if staff receive training, their low retention rate does not allow them to capitalise sufficiently to benefit the RDHA.

##### Supervision of activities by the RDHAs

Participants stressed that RDHA**s** should supervise the Moughataas, their staff, and private (for-profit and not-for-profit) healthcare providers. However, they noted that the integrated supervision of the Moughataas is of poor quality due to the RDHAs’ low competence and insufficient funding and logistics. In addition, supervision reports are very irregular or nonexistent.

*“For some time now*,* there has not been proper supervision. I’m telling you*,* there were heads of health posts who don’t know about RDHA….” (Staff*,* RDHA).*

*“When the RDHA did the supervision*,* he met with all the heads of service in the Moughataa*,* but no reports*,* just a phone call. What is missing*,* in general*,* is the documentation…” (CMOM*).

On the other hand, participants noted that supervision of vertical programs (such as nutrition, medicines, Covid-19, etc.) is done more regularly, as they are funded by partners.

Participants also mentioned that the supervision of RDHA heads of the department is not done internally or at the national level.

*“The RDHA does not formally supervise its heads of department. The staff are left to their own devices… The central level also rarely comes and even if they do it’s quick. Quickly*,* quickly…”. (Staff*,* RDHA).*

The supervision of the private sector is ensured by a general inspector who operates independently and does not report to the RDHA.

##### Planning of activities

Several participants noted that the RDHA should plan its activities on the one hand and support planning at the operational (Moughataa) level on the other. However, they mentioned the RDHA’s weak capacity to do so. They also indicated that for the past two years, annual operational activity plans (OAPs) have been developed with the support of the MOH.

*“Before there was no planning*,* it was rather a “copy and paste” of activities*,* but in any case*,* since the arrival of this ministry*,* we have started with the planning for the year… RDHA organises and informs us*,* sends us the tools beforehand… but RDHA’s capacities are very weak.” (*Chef medical officer of moughataa *“CMOM”)*.

Some participants underlined the RDHA’s low ownership of these OAPs. They also indicated that many of the RDHA’s priority activities included in these OAPs are not funded.

Other participants mentioned that the role of the RDHA would also be to support planning at the operational level. However, this support was also seen as weak and very irregular:

*“Normally the RDHA should support the Moughataa when they do their planning*,* but also in the implementation. But this is not done. It is only when the central level comes…. (Central level).*

#### The operational management

Participants identified the management of human resources (HR) and financial resources (FR), the National Health Information System (NHIS)/epidemiological surveillance, equipment/infrastructure, and medicines/inputs as key functions of RDHA.

##### Human resource management

Several participants noted that the RDHA are supposed to manage (recruitment, retention, motivation, formation, etc.) their HR but also the HR of the Moughataa. They felt that this management was too centralised and inefficient. For example, doctors are assigned directly by the central level to health facilities without going through the RDHA. Paramedics are placed at the disposal of the RDHA, who propose their assignment to the Walis, who are the final decision-makers. Moreover, the reassignment of staff to other regions is done by the central level without consultation of the RDHA, which is only informed a posteriori.

*“The power to manage staff is in the hands of the central level and the Wali*,* not the RDHA. It is the Wali who decides*,* and sometimes politicians force him to favour their relatives. RDHA only proposes*,* it cannot impose. But sometimes it depends on the character and leadership of the RDHA too". (Staff*,* RDHA).*

*“Personnel management is quite centralised. Our opinion is not considered…”. (Staff*,* RDHA).*

As far as the RDHA team is concerned, it consists of six heads of service in addition to the RDHA itself, who is, in general, a medical doctor without any further training. Each service is down to a single head of service, a nurse with no job description and limited organisational capacity and skills.

*“I find that there is a great lack of human resources at the level of the RDHA because you find services with only one staff member. She can’t do the work of a service in the true sense…” (Staff*,* RDHA)*.

In addition, some participants felt that their poor knowledge of public health, particularly of health system management, prevented them from fully playing their roles.

##### Management of financial resources

Some participants noted that financial management was not very effective or efficient. They mentioned that the financing of the RDHA’s activities is essentially provided by the national budget and the TFPs.

*“The RDHA has two sources of funding*,* essentially the Ministry of Health and the partners. But this funding does not arrive in time to carry out the activities” (Staff*,* RDHA).*

The national budget (of the MoH) finances the functioning of the RDHA. While the RDHA formulates requests for disbursements, the Wali is the authorising officer. This reduces the RDHA’s level of accountability. Furthermore, participants noted a long delay in the availability of these resources, sometimes up to six months. This means that RDHAs must go into debt with suppliers who overcharge them to compensate for the delays in payments.

*“It’s complicated if you have a budget of 10 million (MRU)*,* but in fact*,* you only find 5 million. The other 50% goes to the supplier’s percentage and so on…*,* what do you want.” (Staff*,* RDHA).*

As for the TFPs, their funding is centralised at the central level before being transferred to an account managed directly by the RDHA. The RDHA has few partners who directly fund their activities.

*“The partners send their money to the central level and then it is transferred to a bank account at the level of the RDHA to carry out activities such as vaccination campaigns. This works well except that it is often done at the last minute. There is no planning.” (Staff*,* RDHA).*

Furthermore, some participants emphasised that there is little control over the use of financial resources allocated to the RDHA and that the administrative and financial service plays a minor role in their management.

##### Management of the National Health Information System (NHIS) and epidemiological surveillance

For the management of the NHIS, some participants mentioned that the DHIS2 (District Health Information Software) is filled in directly by the Moughataas and that the RDHA no longer has the possibility of validating the data before they are transmitted to the central level, as was the case with the previous software (Maurisys). Therefore, the RDHAs currently play a minor role in the management of the NHIS.

*“Normally*,* it is the RDHAs that collects the data at the regional level*,* it is decentralised. But with the DHIS2 software*,* the Moughataas send the data directly to the Ministry*,* which is a step backwards. It kills the RDHAs…”. (Staff*,* RDHA).*

They mention several problems, such as media stockouts and the poor quality of their data collection and reporting. Despite this, they acknowledge that the NHIS is one of the most successful services because of the pressure exerted by the central level to retrieve data.

*“The NHIS is one of the best services in the RDHA. They are well trained and follow up with the central level daily. It is not bad.” (Staff*,* RDHA).*

In addition, the RDHA coordinates epidemiological surveillance, with daily telephone data collection and transmission to the central level. It also coordinates case investigations as needed with the central and operational levels.

##### Management of medicines and consumables

Participants felt that the RDHAs play a minor role in medicine management. They communicate poorly with the Central Purchasing Office for Essential Medicines and Consumables (CAMEC) and have little power to sanction cases of poor drug management at the operational level.

*“The availability of and access to medicines at the regional level is the responsibility of the RDHA. But it is the CAMEC that manages the* medicines… *We don’t know what’s going on there. We don’t know what happens there. But the RDHAs supervise the pharmaceutical depots in the health posts and centres.” (Central level).*

For the Brakna region, a CAMEC branch located within the RDHA office is responsible for supplying the Moughataas and (regional) hospitals. For Nouakchott-North, supplies are made directly to the central CAMEC or private depots. The role of the heads of the RDHA medicine departments is minor and is limited to supervising the health facilities’ pharmacies to make inventories. This supervision is also irregular.

*“We can’t control what happens to the medicines because the supervision is not regular. There is a lot of mismanagement at the level of the health posts or centres*,* but we don’t have the means to monitor… (Staff*,* RDHA).*

Therefore, the heads of the RDHA services manage only free medicines and consumables to ensure their delivery to the health facilities.

##### Management of equipment and infrastructure

For several participants, the RDHA’s involvement in managing equipment and infrastructure is minor and limited to expressing needs.

*“The RDHA should regularly make an exact situation of the equipment and infrastructure of the Moughataa*,* but nothing is done. Even at the RDHA level*,* there is no up-to-date inventory of materials and equipment.” (Central level).*

In addition, participants indicated that the maintenance of available equipment is not ensured.

*“There is a problem in all the Moughataas for the maintenance of equipment. Normally the RDHA should take care of it. Expensive equipment is bought and then it is not maintained and with small breakdowns*,* it is abandoned. This is not good.” (TFP).*

### Culture and values maintaining: the organizational culture and values of the RDHA

Several participants noted that RDHA’s organisational culture is imbued with Mauritanian social values (practices, habits, or characteristics). As a result, it is not structured or sufficiently formalised and hampers the achievement of the organisation’s objectives.

*“The culture of RDHA is dependent on certain values in society. In Mauritania*,* a lot of things in everyday life are taken lightly and everything is put into perspective… For RDHA*,* this means that many things are done more often in an informal or unstructured way.” (TFP)*.

Some participants see this as an opportunity, as it offers certain flexibility so that, thanks to the reforms underway, the RDHA can develop its own identity and become an organisation that plays its role fully.

On the other hand, some participants refer to certain practices such as clientelistic relationships that guide the organisation of work within the RDHA. Indeed, they mention that some heads of service enjoy more trust from the directors than others. They are therefore given more responsibilities, carry out tasks that should be carried out by their colleagues, or receive benefits, which are sources of frustration.

*“Sometimes the work is distributed among the heads of the department according to the personal relationship with the director. People who are well considered will get more benefits like training and activities… This is not good.” (Staff*,* RDHA).*

Although several participants considered the subject to be sensitive, they felt that ethnicity would influence decisions about appointments to key positions or the allocation of work at the expense of actual skills.

*“Ethnicity interferes with management at the RDHA level. Relatives may be privileged over others and their wrongdoing tolerated. But this is a very sensitive issue in Mauritania…” (TFP).*

Furthermore, the existence of a strong hierarchical culture was also recognised by most participants. However, when the directives given are in contradiction with the regulations and standards, this compromises the achievement of objectives.

*“Staff are expected to carry out the orders of their superior. Sometimes it’s good if it’s part of our job. But sometimes the orders are not correct*,* and the superior imposes himself*,* it influences negatively…” (Staff*,* RDHA).*

On a completely different level, participants mentioned that respect for women and the elderly have implications for the RDHA’s organizational culture. For example, it would not be acceptable to impose a certain work pace on them. However, participants noted that many officers abuse this.

*“We always want to help the elderly or the woman*,* if they have work that is tiring*,* we can take some of it to relieve them.” (Staff*,* RDHA).*

On the other hand, participants indicate that there is a certain amount of multi-tasking of staff which appears to improve their performance. However, some participants acknowledged that this serves the personal interests of staff who are often absent without having to justify themselves. Similarly, it leads to an overload of work for the most conscientious officers.

*“Multi-skilling is compulsory because there are not many of us. If this man is absent or on leave*,* if I know his role*,* it’s better. I can help. But I tell you*,* it depends on the mentality. Some exaggerate and don’t work.”(Staff*,* RDHA).*

### Adaptation to the environment: stakeholder interactions and health coordination

Participants identified two main categories of actors or stakeholders (internal and external) and classified them according to their level of power and legitimacy. They also noted the interactions between stakeholders and their own perceptions of how health action is coordinated at the regional level.

#### Internal actors

These were defined as those who are under the RDHA’s hierarchy and are more closely aligned with its goal:

*“These are the regional hospitals*,* the health facilities and the private healthcare sector (profit and non-profit).”* (Staff, RDHA).

About hospitals, in particular, some participants mentioned inconsistencies in their status that create a source of confusion and sometimes even tensions that would be detrimental to the proper functioning of the RDHA:

*“On the organisational level*,* hierarchically*,* the RDHA is the supervisor of the hospitals at the regional level. The RDHA represents the Ministry of Health. But in addition to that*,* the hospitals have an autonomous management. Moreover*,* the hospitals and the RDHA depend on two different directorates. This creates confusion…” (Hospital Director)*.

*“The problem we have at the RDHA is a problem of institutional anchoring*,* i.e. the RDHA is appointed by our ministry*,* the director of a hospital is appointed by a decree taken in the council of ministers…” (Central level)*.

For several participants, these inconsistencies resulted in weak articulation between the RDHA and the hospitals. However, in the Brakna region, the RDHA is a member of the hospital’s board of directors, which gives it greater visibility. This is not the case for the RDHA in Nouakchott-North.

#### External actors

External actors have been defined as those who are not under the direct hierarchy of the RDHA. These are the central level of the MOH and its attached directorates; the Wilaya and other regional directorates (hydraulics, education, etc.); the TFPs; civil society (NGOs, associations, etc.); the regional health development council (RHDC) or professional orders. Among these, the Wali and the Minister of Health were recognised as the most legitimate and powerfull, which makes them more influential in the functioning of the RDHA.

*“The RDHA is under the direct hierarchy of the Wali (representative of the President of the Republic) and the Directorate General of Health for the MOH. But functionally*,* all the directorates of the ministry must work with the RDHA. So*,* for us*,* the RDHA is the small MOH in each region.” (Central level)*.

As for the TFPs, they are generally aligned with the objectives of the RDHA but can easily influence priorities because of the funding they provide. Their interventions are largely centralised at the level of the MOH with a few exceptions:

*“We don’t have many direct partners. I see ACF supporting us through UNICEF about inputs for nutrition. For the big partners*,* they are managed directly by the ministry.” (Staff*,* RDHA).*

The participants mentioned the role that the RHDC should play in coordinating health action at the regional level. However, these RHDCs are new actors whose capacities are still very weak.

#### Interactions between stakeholders and coordination of regional health action

Several participants agreed that the interactions between stakeholders in health action at the regional level remain weak. This is reflected in the absence of a functional and structured regional consultation framework.

*“Well*,* there is no real coordination of actions except in the case of epidemics such as COVID*,* because it is a national or international problem. It is the Wali who coordinates this with the RDHA…” (Staff*,* RDHA).*

For the participants, this weak coordination is also reflected in the numerous urgent instructions or requests made to the RDHA by the central level and the international partners. They impose on the RDHA the pace of work and priorities.

On the other hand, coordination with the chief medical officers of the Moughataas (CMOM) seems to be more effective, yet is often done by telephone on an adhoc base. Only in Brakna attempts are made to have monthly meetings with the CMOM.

*“We have good coordination with the RDHAs. We can’t go two days without calling each other… Well*,* there is the monthly meeting where all the chief doctors are invited to coordinate together.” (*CMOM*).*

As far as the coordination of activities is concerned, *participants noted that* teamwork is weak and synergies between the heads of services are rare. It is common for the RDHA to approach the heads of departments individually to give them assignments or files according to their affinities, which is often a source of tension.

## Discussion

This study assessed the performance of two regional health departments (RDHA) in Mauritania through an analysis of their functions. The participants noted in particular the lack of clarity in the objectives of the RDHA, the weakness of the services offered, operational management and coordination of health action with the actors (or stakeholders), but also a weak organisational culture. Similarly, the insufficient capacity of the RDHA to maintain a balance between the tensions generated by the interactions between these functions seems to contribute to their poor performance. The main bottlenecks to the performance of RDHAs were found to be related to their weak technical capacities, a rather bureaucratic view of their functions and their unfavourable environment. These bottlenecks appear to be of a magnitude that would not allow the RDHA to fully play its role. They also seem to have been exacerbated by institutional instability due to the recurrent changes in the Minister of Health and his cabinet. Indeed, since 2017, three ministers of health have succeeded each other in Mauritania. In addition, the performance of the RDHA seems to have been affected by the uncertainties created by the opening of the RDHA competition by the MOH for over a year. This has resulted in mistrust and reluctance to take initiatives that may expose them further, and has led to a slowdown in activities.

Furthermore, this study helps to confirm the multidimensional nature of performance for which the analytical framework of Marchal et al. [5] is used. This integrative framework allowed for a systemic analysis of the performance dimensions of the two RDHAs, their interactions and the resulting tensions.

To address the challenges identified, the next section discusses recommendations structured around the four functions and their alignments to initiate an organisational change of the RDHA, thus contributing to improving its performance. This organisational change is essential to the transformation of any organisation and the reform of the health system [16, 17].

Firstly, the study notes the importance of clarifying and sharing the vision and objectives of the RDHA. This could be done under the leadership of the MOH and in alignment with Decree 178/2016/ MOH which states that: *“the RDHAs ensure the implementation of the national health policy at the Wilaya level…”.* These reflections might help to engage all stakeholders and ensure their efforts are complementary towards building the capacity of the RDHA. Similarly, it might help to overcome some of the inconsistencies or overlaps between the different levels of the health system, and also between the heads of the RDHAs themselves. In addition, it might allow for a better alignment of the objectives of the RDHA with the other three functions to facilitate trade-offs and thus minimise possible resistance to change.

Secondly, the participants in the study demonstrate a good knowledge of the services that are supposed to be provided by the RDHA and share the importance of ensuring adequate operational management. However, in reality, the effectiveness and efficiency of these services remain rather limited as was the case in the Democratic Republic of Congo [18]. To achieve the objectives of the RDHAs in Mauritania, it seems important to first revise their current organisational chart to put in place efficient Regional Management Teams (RTMs) capable of steering regional health action. This requires the development of job profiles to guide the selection of RTM members, job descriptions to identify their needs and individual development plans to strengthen their capacities. These RTM members should be trained on their specific needs as a matter of priority to provide quality services. More attention should be paid to the support they should provide to the operational level (Moughataas) which is relegated to the background to respond to the numerous requests from the central level.

Thirdly, the analysis of the environment in which the RDHAs operate has made it possible to identify the main actors in health action at the regional level, among which those at the central/MoH level and the Wali stand out for their power and legitimacy. One striking fact is the lesser attention paid to community participation, although it is explicitly recognised in the national health policy document as a driving force in its implementation [19]. However, our results show that there is a need to strengthen stakeholder engagement and interaction. This is a recognised condition for any organisation to perform well [20]. In this case, RDHAs could be more responsive and create opportunities to interact with other actors. This will help to engage these actors and draw on financial resources, but also on non-tangible resources such as respect, trust, reputation and knowledge that are useful for mobilising them on their behalf. These elements could be facilitated through the creation of a formal and effective coordination mechanism to mobilise actors around regional health action. This coordination could begin, for example, by holding quarterly meetings with the actors directly involved in the provision of care. These meetings could be extended every six months to include the other players in regional health action.

Finally, as suggested by Marchal et al. [5], we sought to identify the main formal and informal values and organisational culture that shape the performance of the RDHA. It was surprising to note that the participants did not explicitly mention certain values that are central to the national health policy for 2030, such as equity, ethics, social justice and solidarity or human rights [19]. Rather, participants referred to wider societal values and interests, that shape the organisational culture of RDHAs and the resulting tensions with certain professional or public service requirements. On the other hand, it should be noted that this is quite common for other organisations in Mauritania or elsewhere in Africa and does not seem to be a problem in the collective imagination. Also, in Africa, such arguments that cut short any criticism are often put forward to try to legitimise or justify the lack of performance of organisations. However, these observations are consistent with other studies which indicate that personal and societal factors determine the culture of organisations. They indicate that clarifying the vision or building the skills to deliver services will not be enough to ensure an organisation’s performance [21–24]. They argue for a comprehensive, sustainable but complex approach to initiating change in the organisational culture of RDHA. This approach needs to involve all stakeholders so that one recognises the benefits of changes to meet their needs and the actions to achieve them are jointly identified. To ensure success, these changes need to be simple, sustained and introduced on a small scale or in stages to be gradually rolled out, reinforced and institutionalised. Indeed, the rapid introduction of change may be counterproductive to the current weak capacity of the RDHAs and may undermine their acceptance and commitment by stakeholders and thus undermine their impact and sustainability. To implement and sustain change, high-level leadership support is essential - for example from the MOH - to provide consistent guidance and facilitate staff engagement and the establishment of communication channels to support dialogue (top down and bottom up) with all stakeholders to have a lasting impact on the change process [16, 25, 26]. Staff engagement can enhance their autonomy and involvement by mobilising a critical mass of support for the process and thus minimise potential resistance to change. The weak anchoring and informal nature of the RDHA’s organisational culture were seen to facilitate these changes [27–30].

## Conclusion

This study identified the main challenges or bottlenecks to the performance of the Brakna and Nouakchott-North RDHAs. They relate to the four functions identified by the Marchal et al. model, which proved to be useful and relevant for this analysis. The main bottlenecks identified relate to the lack of clarity of the RDHA’s objectives, the weakness of the services offered, operational management and coordination of health action with the actors (or stakeholders), but also an organisational culture that is too informal. Similarly, the insufficient capacity of the RDHA to balance the tensions between the interactions between these functions seems to contribute to their poor performance. The scale of these challenges does not allow RDHAs to engage and mobilise the right stakeholders and resources to achieve these objectives. We propose to initiate organisational change at the RDHA level, and some avenues have been discussed. We suggest that they should be studied in greater depth and tested in the context of an (participatory) action research approach to document them and draw lessons for improving the performance of the RDHA in Mauritania. Research on the regional level of the health system in sub-Saharan Africa is very weak and must be developed if it is to play its full role.

## Data Availability

The datasets used and/or analysed during the current study are available from the corresponding author on reasonable request.

## Abbreviations

LMICs: Low- and middle-income countries
SSA: Sub-Saharan Africa
RDHAs: Regional Directorate for Health Action
AI-PASS: (*appui institutionnel au programme d'appui du secteur de la santé*): Institutional support for the health sector support program
ITM: Institut of tropical medicine
MoH: Ministry of health
TFPs: Technical and financial partners
MSACF: Ministry of social affairs, childhood and the family
CMOM: Chef medical officer of moughataa
HR: Human resources
CAMEC: Central purchasing office for essential medicines and consumables
RHDC: Regional health development council
RTM: Regional management teams
ACF: Action contre la faim
